# Managing Injuries of the Neck Trial (MINT): design of a randomised controlled trial of treatments for whiplash associated disorders

**DOI:** 10.1186/1471-2474-8-7

**Published:** 2007-01-26

**Authors:** Sarah E Lamb, Simon Gates, Martin R Underwood, Matthew W Cooke, Deborah Ashby, Ala Szczepura, Mark A Williams, Esther M Williamson, Emma J Withers, Shahrul Mt Isa, Anil Gumber

**Affiliations:** 1 Warwick Clinical Trials Unit, Warwick Medical School, University of Warwick, Coventry CV4 7AL, UK; 2 Kadoorie Critical Care Research Centre, John Radcliffe Hospital, Oxford, OX3 9DU, UK; 3 Centre for Health Sciences, Abernethy Building, 2 Newark Street, Barts and The London, Whitechapel, London E1 2AT, UK; 4 Wolfson Institute of Preventive Medicine, Barts and The London, Queen Mary's School of Medicine & Dentistry, University of London, Charterhouse Square, London EC1M 6BQ, UK; 5 Centre for Evidence in Ethnicity, Health and Diversity, Warwick Medical School, University of Warwick, Coventry CV4 7AL, UK

## Abstract

**Background:**

A substantial proportion of patients with whiplash injuries develop chronic symptoms. However, the best treatment of acute injuries to prevent long-term problems is uncertain. A stepped care treatment pathway has been proposed, in which patients are given advice and education at their initial visit to the emergency department (ED), followed by review at three weeks and physiotherapy for those with persisting symptoms. MINT is a two-stage randomised controlled trial to evaluate two components of such a pathway: 1. use of The Whiplash Book versus usual advice when patients first attend the emergency department; 2. referral to physiotherapy versus reinforcement of advice for patients with continuing symptoms at three weeks.

**Methods:**

Evaluation of the Whiplash Book versus usual advice uses a cluster randomised design in emergency departments of eight NHS Trusts. Eligible patients are identified by clinicians in participating emergency departments and are sent a study questionnaire within a week of their ED attendance. Three thousand participants will be included. Patients with persisting symptoms three weeks after their ED attendance are eligible to join an individually randomised study of physiotherapy versus reinforcement of the advice given in ED. Six hundred participants will be randomised. Follow-up is at 4, 8 and 12 months after their ED attendance. Primary outcome is the Neck Disability Index (NDI), and secondary outcomes include quality of life and time to return to work and normal activities. An economic evaluation is being carried out.

**Conclusion:**

This paper describes the protocol and operational aspects of a complex intervention trial based in NHS emergency and physiotherapy departments, evaluating two components of a stepped-care approach to the treatment of whiplash injuries. The trial uses two randomisations, with the first stage being cluster randomised and the second individually randomised.

## Background

Whiplash injuries are a major health and economic problem around the world. In the UK, their annual cost to the economy is about £2,553 million (1990 prices), representing about 18% of the total costs of all road traffic collisions and 0.4% of the Gross Domestic Product[1]. The costs are caused by absence from work due to injury and considerable health service costs. Most patients recover quickly but a substantial proportion, estimated by different studies at between 19% and 60%[2,3], may develop chronic symptoms. These patients generate the majority of costs, and prevention of chronic symptoms is therefore a priority in treatment of whiplash injuries.

Definitions of whiplash and associated conditions vary between published studies. The Quebec Task Force (QTF) definitions are internationally recognised and are used in this study[4]. Whiplash is the mechanism of injury (acceleration-deceleration injuries usually in the frontal plane), whiplash injuries are the soft tissue injuries that result and Whiplash Associated Disorder (WAD) describes the pattern of symptoms that arise (Table 1). A further term, late whiplash syndrome, is used to describe the chronic complications of whiplash.

Despite whiplash being a common injury, there are few good quality randomised trials upon which to base recommendations for practice[5]. In the mid 1990s the QTF undertook an extensive review and expert consensus exercise[4], and found that there was insufficient evidence supporting the treatments currently used. They concluded that promoting activity in the early stages was probably the most effective strategy, soft collars were not helpful, and physiotherapy, a very common treatment, required rigorous evaluation. The QTF proposed a clinical pathway in which patients are given advice and education at the initial contact, and then reviewed at three weeks. Patients with persisting symptoms would then be provided with more intensive treatment.

A stepped care clinical pathway as proposed by the QTF is the model evaluated in this trial. The advice and education component to be evaluated is The Whiplash Book[6]. This has been developed largely based on the results of a systematic review[7], which suggested that psychological risk factors are the strongest predictors of poor outcome in whiplash patients, and argued that advice to resume normal activity, using a cognitive-behavioural approach, should be the treatment of choice for early management. However, other systematic reviews have suggested that physical and psychological factors may carry equivalent amounts of risk for poor outcome[8]. Furthermore, most of the literature cited to support the early activity and key health promotion messages was from the field of low back pain and other chronic conditions. It is questionable whether these results are transferable to acute whiplash injuries, as the conditions differ markedly in their causes and psychological consequences. For example, phobic travel anxiety and other psychological manifestations of shock are common after whiplash[9], but rarely occur with low back pain.

The second component of the stepped care approach evaluated in MINT is physiotherapy for patients whose symptoms have not resolved by three weeks. Physiotherapy treatments that are commonly used for whiplash patients include hot and cold therapy, electrotherapies, mobilisation, manipulation, exercises of many different kinds, and traction. There is good quality trial evidence to support the effectiveness of mobilisation and exercise in the management of chronic neck pain[10-12], but it is uncertain whether these treatments are effective for whiplash patients. The Cochrane review of conservative treatments for whiplash[4] concluded that there was some evidence that active treatments are superior to passive, though the existing trials were not of high quality. Another review concluded that there was moderate quality evidence that exercises and mobilisations commonly used by physiotherapists were effective[13], but this was based on just three small trials, which reported short-term outcomes only, did not perform intention to treat analyses, and did not have blinding of outcome assessment. For evaluation in MINT we have designed a package of physiotherapy treatments that, according to current evidence, are those most likely to be effective in prevention of late whiplash syndrome and to be acceptable to practitioners.

### Current practice

A national survey of practice in the UK indicated that the most common treatment for whiplash in emergency departments (ED) is advice, but the content and quality of the advice varies [unpublished data]. Over 90% of departments suggest using analgesics and gradually increasing movement of the neck. Some departments use soft collars as well, suggesting that they should be removed and the neck exercised on a regular basis.

## Methods

MINT is a multi-centre randomised controlled trial to estimate the clinical effectiveness of a stepped care approach to whiplash injuries on clinical outcomes over 12 months, the effectiveness in pre-specified sub-groups of patients (those with severe physical symptoms, prior neck problems, psychological or physical risk factors for poor outcome, and those seeking compensation), and the costs and cost-effectiveness of each strategy.

The trial will use two separate randomisations: the first stage is a cluster randomised trial in which NHS Trusts are randomised to use the Whiplash Book or give their usual advice, for all patients presenting with whiplash injuries. The second stage is individual randomisation to physiotherapy or the control intervention of a single advice session reinforcing the advice given in ED, for patients still experiencing whiplash symptoms at three weeks. The two parts of the trial have a common system of follow-up at four, eight and 12 months.

The trial is being run in 12 NHS Acute Trusts in the UK: Heart of England NHS Foundation Trust (Heartlands and Solihull Hospitals), North Bristol NHS Trust (Frenchay Hospital), Oxford Radcliffe Hospitals NHS Trust (John Radcliffe Hospital), University Hospitals Coventry and Warwickshire NHS Trust (Walsgrave Hospital and Hospital of St Cross, Rugby), Gloucestershire Hospitals NHS Trust (Cheltenham and Royal Gloucester Hospitals), South Warwickshire General Hospitals NHS Trust (Warwick Hospital), Worcestershire Acute Hospitals NHS Trust (Alexandra Hospital, Redditch), University Hospitals Birmingham NHS Trust (Selly Oak Hospital), Kettering General Hospital NHS Trust (Kettering General Hospital), Buckinghamshire Hospitals NHS Trust (Stoke Mandeville Hospital, Countess of Chester Hospital NHS Foundation Trust (Countess of Chester Hospital), and Gwent Healthcare NHS Trust (Royal Gwent Hospital, Newport). Some Trusts comprise several hospitals and have more than one Emergency Department.

### Ethics Committee approval

MINT was approved by the Trent Multicentre Research Ethics Committee and by the Local Research Ethics Committee and the Research and Development Committee of each participating centre.

### Stage 1: Cluster randomised trial of the whiplash book versus usual advice

#### Inclusion and exclusion criteria

All people who attend ED with a whiplash injury of less than six weeks duration will be included in the trial, except those with any of the following exclusion criteria:

1. Age less than 18 years.

2. Fractures or dislocations of the spine or other bones.

3. Head injuries with more than a transient loss of consciousness or with a Glasgow Coma Score of 12 or less at any stage of their assessment in hospital.

4. Admission to in-patient services.

5. Severe psychiatric illness.

#### Identifying participants and consent

Because the first part of the trial is cluster randomised, individual consent for participation is not sought. This is an accepted procedure for cluster randomised trials where individuals do not have a choice of whether to receive the trial intervention[14]. All eligible patients at each participating hospital are included unless they indicate that they do not wish to participate in data collection.

Clinicians in ED are responsible for identifying eligible participants. Details of whiplash patients are recorded on the trial proforma, a short form developed specifically for MINT that replaces the normal methods of clinical data collection in participating centres. It is intended to avoid duplication of recording of information for clinical and research purposes, and hence allows collection of a routine core clinical data set, including injury severity, pain intensity and WAD grade diagnosis. It contains tick boxes to ensure that clinicians have provided potential participants with the trial information sheet and have discussed the study with them, and also records if the patient would prefer not to receive the study questionnaires. The proforma is self-copying; one copy is filed in the medical notes as a treatment record and the second copy is passed to the research team to notify them that a patient has been asked to participate. Completed proformas are collected in a secure place in the ED and forwarded to the MINT research team twice a week.

Patients are informed about the possibility that they may be eligible for stage 2 of the study but detailed information about this is not given at this stage, as the majority of patients who participate in stage 1 will not have persistent symptoms at 3 weeks and hence will not be eligible for stage 2. Patients are also asked for their contact details (address, phone number, mobile phone and email), to assist with sending out and following up questionnaires.

#### Randomisation

The unit of randomisation is the NHS Trust. Participating Trusts were randomised before the start of recruitment by the project statisticians, to usual advice or the Whiplash Book. Trusts were pair matched on size (number of ED attendances per year), star rating, and ethnic composition of the surrounding area. We randomised by Trust rather than by ED to avoid contamination when staff of one Trust worked in more than one ED. Randomisation used a table of random numbers, starting at a random place to ensure that the allocations were not known before randomisation. The allocation depended on whether the next digit was even or odd. One of each pair was randomised to the Whiplash Book, and the other member was allocated to usual advice.

#### Delivery of interventions

Training of ED staff in the trial procedures is given before the start of recruitment, and there is frequent contact between the centres and the trial team to identify and resolve any problems. Eligible patients are given a letter of introduction about the study, signed by their local ED consultant, and the study is discussed with them. If they are willing to participate, they are told that they will receive a questionnaire in a few days time. They are asked to return this and to contact the MINT study team if they continue to have problems after two weeks. The introduction letter does not mention randomisation of hospitals to The Whiplash Book or usual advice, but simply states that the hospital is taking part in a study of advice given to patients with whiplash injuries. ED clinicians provide a copy of either the ED's usual advice leaflet or the Whiplash Book, and verbal guidance on management of whiplash injuries. We have obtained copies of the usual advice leaflets from all of the EDs participating in MINT, so that the content of the advice in the control arm can be documented.

#### Baseline data collection

All whiplash patients that are eligible for MINT and have not asked to be excluded are sent a copy of the MINT baseline questionnaire within a week of their ED attendance. This includes demographic information and baseline administration of some of the outcome measures. If the questionnaire is not returned within a week, participants are sent a reminder by SMS text message, email or post.

### Stage 2: Individually randomised trial of physiotherapy versus reinforcement of advice given in ED

#### Identifying participants and consent

Participants in Stage 1 are asked to contact the study office if they continue to have symptoms two weeks after their attendance at ED. An appointment is then made for the patient with a research physiotherapist based at their local hospital. At this appointment, their eligibility for Stage 2 of the trial is assessed. If eligible, trial participation is discussed and the patient is asked to sign a study consent form prior to randomisation. Information about Stage 2 of MINT is sent to patients several days before their research clinic appointment, ensuring that they have sufficient time to consider participation.

#### Inclusion and exclusion criteria

Participants in Stage 1 of MINT are eligible for the second part of the trial if they:

• Report symptoms in the 24 hours before attendance at the physiotherapy research clinic approximately three weeks after attendance at ED

• Are WAD grade I-III at this time

• Do not have any contra-indications to physiotherapy treatment. These include central cord compression or upper motor neuron lesion, complete nerve root compression or lower motor neuron lesion, suspected vascular injury or haemorrhagic event.

#### Randomisation

Randomisation to physiotherapy or reinforcement of advice is via a central telephone randomisation service, based at the Cancer Research Clinical Trials Unit, University of Birmingham. Randomisation is stratified by centre, to avoid imbalance between centres giving different advice in ED, and members of the same household are assigned to the same intervention, to reduce the chance of contamination. This will be taken into account in the trial analysis. If eligible patients decline participation, their reasons for doing so are recorded.

#### Interventions

All interventions are delivered by physiotherapists who are independent of the recruitment and randomisation procedures, and have attended a 1.5 day training session by the trial team. The same therapists deliver both the physiotherapy and control interventions, and each treatment session is recorded in a treatment log. A sample of sessions of both interventions is observed for quality control purposes. All treatments should be completed within four months of the patient's first attendance at ED.

(a) Physiotherapy

Participants who are randomised to the physiotherapy package have up to six sessions of therapy, over an eight week period. The components of the intervention are described in a training and reference manual. The choice of physiotherapy treatments has been made using two principles; first, there is evidence that the treatments are effective for chronic neck dysfunction and are likely to be effective for whiplash injuries, based on expert opinion or limited trial evidence, and second, the treatments target established and potentially modifiable risk factors for developing late whiplash syndrome, including reduced cervical range of motion, high pain intensity, and adverse psychological reactions to the injury.

Three treatments are included in the physiotherapy package:

(1) Mobilisation (gentle manipulation) of the cervical and upper thoracic spine according to Maitland[15].

(2) Exercises for the cervical spine, thoracic spine and shoulder to improve range of movement and muscle control.

(3) A cognitive behavioural approach to treatment delivery, which has been effective in physiotherapy for other painful conditions[16].

Manipulation (Maitland Grade IV) of the cervical spine is excluded from this treatment package. Both whiplash injury and cervical manipulation have the potential to cause damage to the vertebral artery that may result in a cerebrovascular event. In common with some, but not all, authorities we consider that recent trauma is a contraindication to cervical manipulation.

(b) Reinforcement of advice

Participants randomised to reinforcement of advice receive a single 40-minute session of advice from a physiotherapist. At this session, the physiotherapist re-states the advice that the patient was given at the time of their ED attendance (either the Whiplash Book or the hospital's usual advice), discusses any queries that the patient may have, and may check the exercises that the patient was given in ED. The physiotherapist can only give advice regarding progression of exercises or activities specified in the Whiplash Book or usual advice. They cannot prescribe new exercises or use any "hands on" treatment. No review appointments are offered to these patients. They are advised to see their GP if they have ongoing problems.

### Other treatments

Participants may seek other forms of treatment during the follow up period from their GP or other health professionals. If the trial interventions are effective, this should be evident in a reduction in additional treatments. Such treatments, including changes in the amount or types of analgesia used, use of physical treatments (osteopathy, chiropractic or physiotherapy), alternative therapies, or referral to secondary care services will be recorded as a treatment outcome.

### Outcome measures and data collection

Follow-up data collection is by postal questionnaire. The outcome measures are detailed in Table 2. The primary outcome is return to normal function after the whiplash injury, measured using the Neck Disability Index (NDI). The NDI is a self-completed questionnaire that has been used successfully in a postal format in trials of neck treatments[10,17]. It assesses pain-related activity restrictions in 10 areas including personal care, lifting, sleeping, driving, concentration, reading and work. The SF-12 and EQ-5D are included to assess generic health-related quality of life, and to enable a single utility score for economic evaluation to be derived from the EQ-5D. Participants also rate whether they have improved, remained the same, or worsened, and their satisfaction with treatment. Resource use is assessed by a short questionnaire which asks about additional NHS or private hospital treatment for the whiplash injury, any GP consultations, manipulation, massage or other treatment. Participants are asked to distinguish between prescription and out-of-pocket expenses.

Participants are asked at 12 month follow-up whether they have pursued and settled a compensation claim related to their whiplash injury. It is not asked at 4 or 8 month follow-up to avoid stimulation of claims among the trial population.

A research assistant who has not been involved in the recruitment or randomisation processes is responsible for mailing follow up questionnaires, and for entering responses onto the study database. Blinding of the study team will be maintained until final analysis of the data has been completed.

### Statistical analysis

The analysis will be by intention to treat. All patients will be analysed in the groups to which they were randomised, regardless of the treatment that they actually received. The two main comparisons will be Whiplash Book versus usual advice, and physiotherapy package versus reinforcement of ED advice. The comparison of ED advice interventions will use appropriate methods to take account of the cluster randomisation[18]. Estimates of treatment effect with 95% confidence intervals, and the numbers needed to treat, will be reported. Additional exploratory analyses will investigate whether there is an interaction between the ED advice intervention and physiotherapy.

Four pre-specified subgroup analyses will be undertaken:

1. severe physical symptoms at trial entry (WAD Grade III versus WAD Grade I or II)

2. adverse psychological reactions at trial entry (yes/no)

3. pre-existing neck pain versus no pre-existing neck pain

4. compensation; claim being pursued versus not being pursued

Statistical tests of interaction will be used to perform subgroup analyses[19].

Economic analysis will use cost-minimisation, or cost-effectiveness and cost-utility analysis, depending on the clinical results. For cost-utility analysis, the EQ-5D will be used to generate utility scores, which will provide an estimate of the incremental cost of any benefit gained in terms of improved health status. Decision modelling will be used to investigate the costs and benefits of the different patient management routes, and uncertainty will be quantified by multi-way sensitivity analyses[20].

### Sample size

For the primary outcome of NDI, there is consensus that a minimal clinically importance difference lies in the range of 3–5 percentage points, with a standard deviation of about 8%[8]. We therefore aim to be able to detect a difference between the groups of three percentage points (i.e. 0.375 standard deviations), both for the comparison of the Whiplash Book and usual advice, and for physiotherapy versus reinforcement of advice. For the individually randomised comparison (physiotherapy versus reinforcement of advice), 211 per group will be required, based on 90% power and 1% significance level. Assuming a worst case scenario of 30% loss to follow-up gives a total sample size of 300 per group (600 in total)[21]. The comparison of ED advice interventions is cluster randomised, so larger numbers are needed. Originally it was planned that eight centres would participate, recruiting 4,800 participants. This was revised with the inclusion of four additional centres, which allowed reduction of the overall sample size required to achieve the same power. Assuming an intra-cluster correlation co-efficient of 0.02, and an average of 120 patients per centre gives an inflation factor of 5.94[22], leading to a sample size of 713 in each group. Allowing for 30% loss to follow up, 1020 participants per group will be needed (2040 in total). To allow for a reduction in power caused by unequal sample sizes among clusters, the target sample size has been set to 3000 (an average of 250 per cluster). The assumptions underlying the sample size calculation will be monitored by the DMEC during recruitment and adjustments may be made during the course of the trial.

## Competing interests

The author(s) declare that they have no competing interests.

## Authors' contributions

SEL designed the study, secured funding and contributed to writing the paper.

SG contributed to protocol revisions and drafted the paper.

MRU contributed to design of the study and securing funding and revised the paper.

MWC contributed to design of the study and securing funding and revised the paper.

DA was responsible for statistical aspects of study development, contributed to securing funding and revised the paper.

AS was responsible for economic aspects of study development, contributed to securing funding and revised the paper.

MAW was responsible for the clinical trial co-ordination and design of the intervention.

EMW was responsible for assisting with the clinical trial co-ordination and design of the intervention.

EJW was responsible for the trial co-ordination, recruitment, data cleaning and management.

SM was responsible for statistical analyses of the study and development and maintenance of the study databases.

AG was responsible for economic analyses of the study.

All authors read and approved the manuscript.

## Pre-publication history

The pre-publication history for this paper can be accessed here:



## Figures and Tables

**Table 1:** Case definitions of Whiplash Associated Disorders

**Term**	**Definition**
WAD Grade 0	No neck complaints or signs
WAD Grade I	Complaint of pain, stiffness or tenderness, but no physical signs
WAD Grade II	Complaint of pain, stiffness or tenderness, and musculo-skeletal signs (decreased range of motion, point tenderness etc)
WAD Grade III	Complaint of pain, stiffness or tenderness and neurological signs (decreased or absent deep tendon reflexes, weakness and sensory deficits). Could also have musculo-skeletal signs.
WAD Grade IV	Fracture or dislocation
Late whiplash syndrome	Presence of pain, restriction of motion or other symptoms at six months or more after the injury, sufficient to hinder return to normal activities such as driving, usual occupation and leisure.

**Table 2:** Outcome measures

Domain	Measures*	Time points
Function	Neck Disability Index	4, 8, 12 months
	Time to return to work and normal activities (including driving)	4, 8, 12 months
Health-related quality of life	SF12	0, 4, 8, 12 months
Satisfaction	5 point Likert scale	4, 8, 12 months
Economics	Resource use questionnaire	0, 4, 8, 12 months
	EQ-5D (health utility)	0, 4, 8, 12 months

*all outcome measures are collected by self-completed postal questionnaires
